# Supplementary material for: Slow Release and Water Retention Performance of Poly(acrylic acid-co-acrylamide)/Fulvic Acid/Oil Shale Semicoke Superabsorbent Composites
Source: Polymers (Basel). 2022 Apr 22;14(9):1719. doi: 10.3390/polym14091719 (PMC9104033; doi:10.3390/polym14091719)
Supplement: Supplementary file 1 [file polymers-14-01719-s001.zip › polymers-1690353-SM.pdf]

Slow release and water retention performance of poly(acrylic acid-*co*-acrylamide)/fulvic acid/oil shale semicoke superabsorbent composites

Yongsheng Wang [1,2], Yongfeng Zhu [1,*], Yan Liu [1], Bin Mu [1], Aiqin Wang [1,*]

[1] *Key Laboratory of Clay Mineral Applied Research of Gansu Province, Center of Eco-material and Green Chemistry, Lanzhou Institute of Chemical Physics, Chinese Academy of Sciences, Lanzhou 730000, P.R. China*

[2] *Center of Materials Science and Optoelectronics Engineering, University of Chinese Academy of Sciences, Beijing 100049, P.R. China*

[*]Correspondence to: Aiqin Wang (E-mail: aqwang@licp.cas.cn), Tel: +86-931 4968118, Fax: +86-931 4968019.

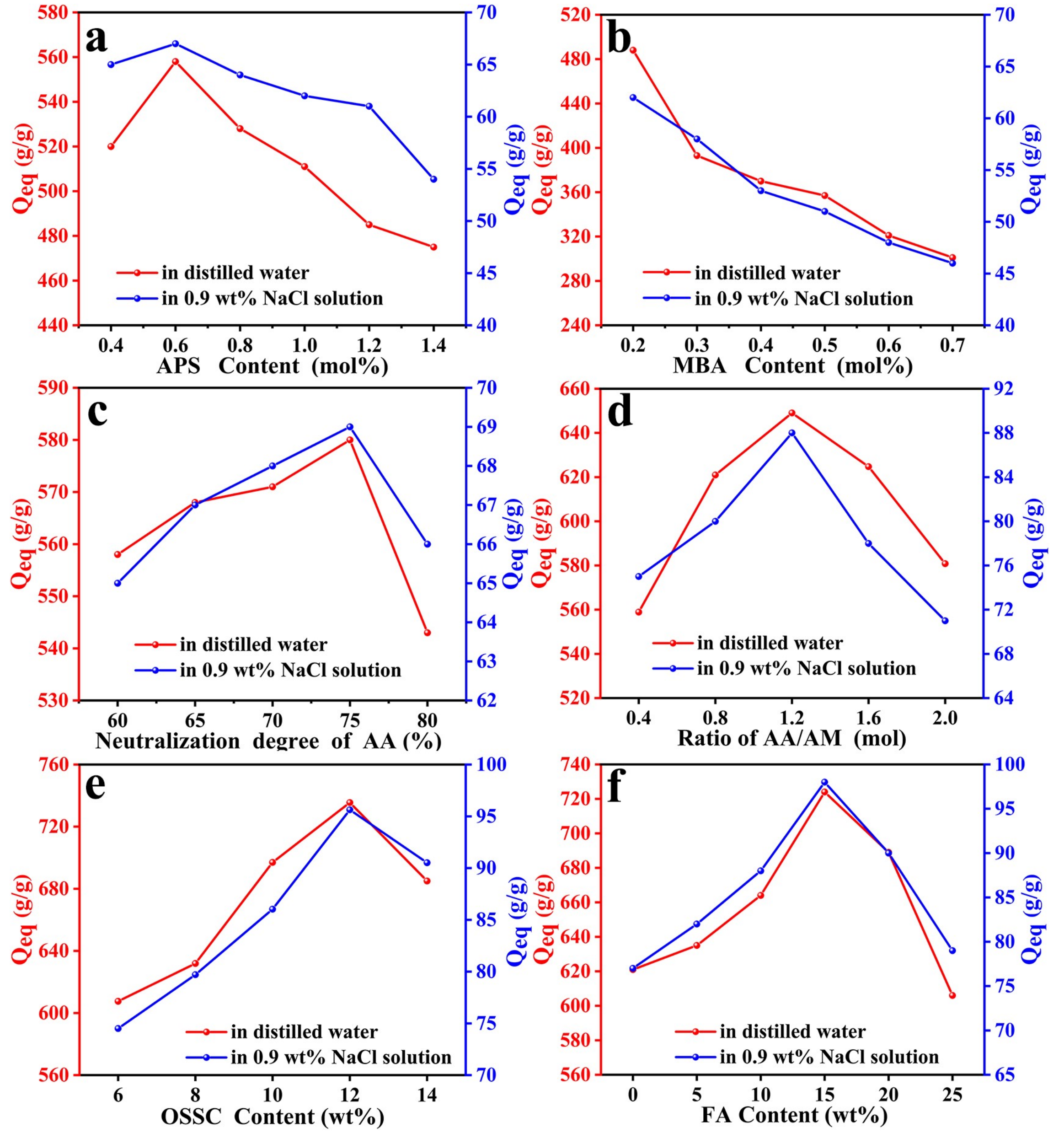

Figure S1. The effects of synthesis conditions on water (salt) absorbency of PAMFS: Effect of APS content (a), MBA content (b), the neutralization degree of AA (c), the ratio of AA/AM (d), OSSC content (e) and FA content (f).

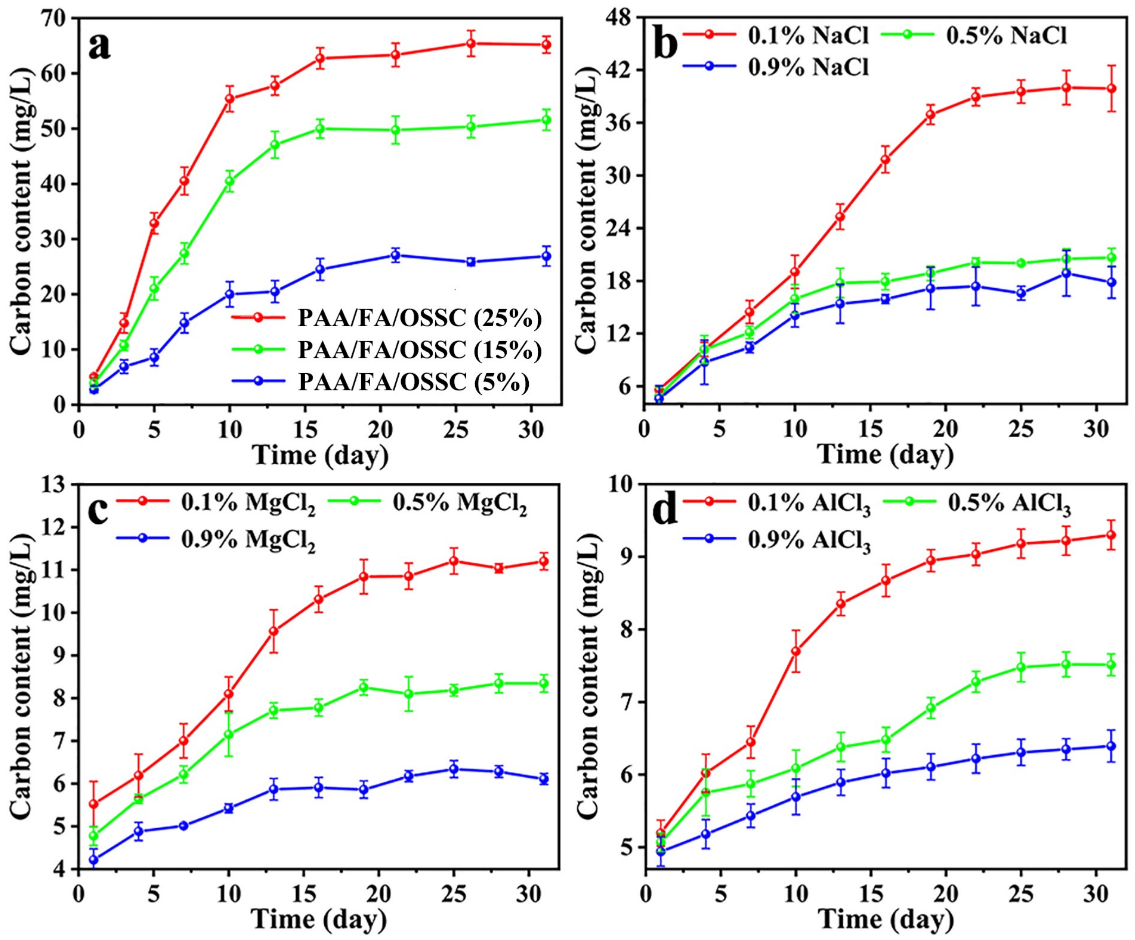

Figure S2. FA slow release of PAA/FA/OSSC in distilled water (a); FA slow release of PAA/FA/SC (15%) in NaCl salt solution (b), $MgCl_2$ salt solution (c), and $AlCl_3$ salt solution (d).

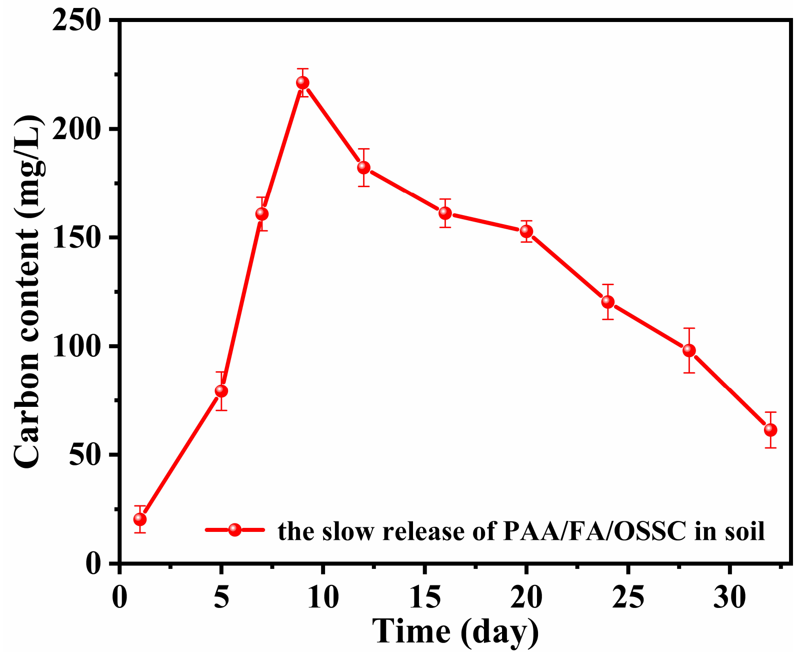

Figure S3. The slow release of PAA/FA/OSSC in soil.

Table S1. Comparing with the references on slow-release fertilizers including NPK and HA in recent years

Slow-release material	Swelling capacity		Time for maximum sustained release accumulation		References
	in distilled water	in 0.9 wt% NaCl solution	in distilled water	in soil	
P(AA-co-AM)/FA/SC	724 g/g	98 g/g	16 d	20 d	Current research
PAA/HA/SC	824 g/g	80 g/g	15 d	9 d	[7]
MOF MIL-100(Fe)@CNFs	37 g/g	--	10 h	--	[8]
SDB-pellet	--	--	10 d	20 d	[9]
NPK/CS-Gl-K	90 g/g	--	20 d	--	[10]
CSt-g-PAA/NR/PVA	321 g/g	57 g/g	60 h	20 d	[11]
Slow-release fertilizers (WSF)	178 g/g	48 g/g	6 h	5 d	[12]
Starch-based superabsorbent polymers (SBSAPs)	253 g/g	57 g/g	20 d	--	[13]
Wheat nano-biochar (WBNC)	98 g/g	--	10 d	18 d	[14]
PAA/AMPS/CTS/SH	998 g/g	101 g/g	10 d	--	[15]
SBS-g-P(AA/AM)-UF	109 g/g	43 g/g	--	10 d	[16]
CSGCHs	298 g/g	47 g/g	120 h	--	[17]

Table S2. The swelling kinetic parameters of PAMFS and PAMS in distilled water or 0.9 wt% NaCl solution.

Samples	The swelling kinetic parameters					
	in distilled water			in 0.9% NaCl solution		
	Q_∞ (g/g)	K_s ($\times10^{-5}$, g/g·s)	n	Q_∞ (g/g)	K_s ($\times10^{-5}$, g/g·s)	n
PAMFS	729.93	0.4066	0.4679	105.93	2.9814	0.4024
PAMS	632.91	0.2958	0.5214	75.36	3.1573	0.4217
